# Bearing Fault Diagnosis of Hot-Rolling Mill Utilizing Intelligent Optimized Self-Adaptive Deep Belief Network with Limited Samples

**DOI:** 10.3390/s22207815

**Published:** 2022-10-14

**Authors:** Rongrong Peng, Xingzhong Zhang, Peiming Shi

**Affiliations:** 1 Nonlinear Dynamics and Application Research Center, Nanchang Institute of Science and Technology, Nanchang 330108, China; 2 National Engineering Research Center for Equipment and Technology of Cold Rolled Strip, Yanshan University, Qinhuangdao 066004, China; 3 College of Electrical Engineering, Yanshan University, Qinhuangdao 006004, China

**Keywords:** rolling mill, rearing fault diagnosis, 2D spectral kurtosis image, ISSA-DBN, limited samples

## Abstract

Given the complexity of the operating conditions of rolling bearings in the actual rolling process of a hot mill and the difficulty in collecting data pertinent to fault bearings comprehensively, this paper proposes an approach that diagnoses the faults of a rolling mill bearing by employing the improved sparrow search algorithm deep belief network (ISAA-DBN) with limited data samples. First, the fast spectral kurtosis approach is adopted to convert the non-stationary original vibration signals collected by the acceleration sensors installed at the axial and radial ends of the rolling mill bearings into two-dimensional (2D) spectral kurtosis time–frequency images with higher feature recognition, and the principal component analysis (PCA) technique is used to decrease the dimension of the data in order to achieve a high diagnosis rate with a limited number of samples. Subsequently, the sparrow search algorithm (SSA) is used to realize the intelligent optimized self-adaptive function of a deep belief network (DBN). Furthermore, the firefly disturbance algorithm is employed to improve the spatial search capability and robustness of SSA-DBN in order to achieve better performance of the ISSA-DBN method. Finally, the proposed approach is experimentally compared to other approaches used for diagnosis. The results show that the proposed approach not only retains the useful features of the data through dimension reduction but also improves the efficiency of the diagnosis and achieves the highest diagnosis accuracy with limited data samples. In addition, the optimal position of the sensor for diagnosing rolling mill roll faults is identified.

## 1. Introduction

The strip mill is the main piece of equipment in the iron and steel industry. It is a highly automated system with a complex structure, and its running health determines its rolling speed and the quality of the rolled products. As the rolling speed and strength continue to increase, the rolling mill equipment suffers from frequent failures, which not only pose a serious threat to the safety and normal production of the rolling production line but also cause significant economic losses. Therefore, monitoring and diagnosing the health status of the key equipment of the hot rolling mill has emerged as an urgent scientific problem [1,2].

Research on the health status of rolling mill equipment requires not only deep theoretical knowledge but also high-precision equipment fault diagnosis technology to identify the causes of equipment faults and provide effective solutions. Initially, researchers had established a flutter mechanical model to study the relationship between abnormal vibrations of rolling mills and equipment failure. Monaco [3] conducted a long-term tracking test on the working condition of a 2030 mm hot rolling mill, established a vibration model by combining electrical and mechanical systems, and obtained the relationship between the amplification coefficient of the workpiece deformation fault and the torsional vibration of the rolling mill. Roberts [4] suggested that transverse stripes on the surface of a workpiece are caused by the rebound of the work roll to its backup roll. Furthermore, he established a mathematical model to predict the resonance frequency and proposed that the rolling mill vibration can be suppressed by appropriately changing the roll speed. Yarita [5] found that abnormal vibration faults of a rolling mill are closely related to the rolling speed, strip thickness, characteristics of the lubrication oil, and other factors by analyzing the measured data on the rolling site. These studies have promoted research on abnormal vibrations of rolling mills and rolling mill equipment faults. However, they mainly deal with rolling mill equipment failure based on the phenomena after the failure. They cannot meet the requirements of on-site rolling process monitoring and diagnosis, and their fault diagnosis performance and identification performance are not ideal.

Since the 1980s, the development of signal processing technology has promoted the rapid development of fault diagnosis technology, and researchers have applied it to the iron and steel industry. In particular, it has been widely used in the detection, identification, and prevention of rolling mill equipment fault signals. Kimura et al. [6] realized a new lubrication system that can improve the rolling speed of thin strip steel and prevent severe chatter faults. Lee et al. [7] used the fast Fourier transform (FFT) to diagnose eccentric faults in the work roll of a rolling mill, thereby reducing the adverse impact of work roll defects on the strip steel quality. He et al. [8] proposed a novel vibration signal detection method for extracting the characteristics of weak chatter faults of the rolling mill generated by chatter traces on the surface of strip steel. Shao et al. [9] proposed an approach for recognizing chatter traces on the surface of strip steel using a kurtosis probability density function and achieved good results through industrial application. Rothera et al. [10] applied the wavelet transform and empirical mode decomposition methods to hot rolling strip data in order to detect the factors affecting the strip quality. Chen et al. [11] proposed a maximum-overlap multi-wavelet denoising method to identify composite faults in the rolling mill reducer. Yuan et al. [12] proposed a technique for diagnosing faults by adopting multi-wavelet sliding window neighborhood coefficient denoising, which can efficiently derive the fault attributes of the main transmission gearbox of the rolling mill. The aforementioned studies used various approaches pertinent to modern signal processing in order to derive and analyze the fault attributes of rolling mill equipment. However, owing to the complex working conditions of the strip mill and several interference factors, the extraction and identification of the fault types and features of rolling mill equipment require further investigation.

The rapid development of artificial intelligence technology has contributed to advancements in mechanical fault diagnosis technology. In particular, since the development and progress of deep learning theory [13], new ideas have been developed for theoretical studies, technical methods, and testing techniques for diagnosing faults of rolling mill equipment. Some studies have examined the condition monitoring and fault diagnosis of rolling mill equipment using deep learning theory. To explore and understand the factors and conditions underlying rolling mill chatter, Perez et al. [14] used an automatic algorithm to extract the dynamic behavior during normal operation as well as chatter faults from a large amount of real data. Moreover, they used visualization technology to provide an interactive interface for effectively displaying the mechanism of mill chatter. Takami et al. [15] applied principal component analysis (PCA) to multi-dimensional data in order to identify faults in the rolling process of a rolling mill. The results showed that their approach can reduce the occurrence of strip defects in the rolling process. Arinton et al. [16] proposed a dynamic high-order neural network with good modeling characteristics, which can effectively identify and robustly detect faults and tension in mill stands. Serdio et al. [17] proposed a residual-based fault detection method that validated three different test scenarios of a steel rolling mill. Xu et al. [18] proposed transfer convolutional neural networks using fault diagnosis online in order to achieve the required fault diagnosis accuracy within limited training epochs and adopted this approach for the fault detection of a rolling mill bearing housing. Zhao et al. [19] combined the adaptive multi-variate variational mode decomposition method with a convolution neural network model to derive fault information based on vibration signals of rolling mill multi-row bearings. Compared to the available approaches in the literature, the accuracy of diagnosing faults for rolling mill bearings using this approach is improved when unbalanced data are encountered. Shi et al. [20] proposed a novel multi-source sensor fusion method that monitors the health status of rolling mills. Although studies on rolling mill equipment fault diagnosis using deep learning theory have achieved some success, most of them are based on a common assumption: that the marked data are sufficient and contain complete information on the health status of the rolling mill equipment. However, in practice, this assumption is unrealistic, because the data collected from the field rolling process have two characteristics. (1) It is difficult for such data to contain sufficient information to reflect the integrity of the health and fault status of the rolling mill equipment. Because most rolling mill equipment operates in a healthy state and faults seldom occur, it is easier to collect health data than fault data, which will lead to incomplete data collection. (2) Most of the collected data are unlabeled because it is unrealistic to stop frequently to check the health status, which is time-consuming and will lead to economic losses. Accordingly, it is necessary to develop a more reliable model to diagnose faults of rolling mill equipment when limited samples are available.

In summary, thus far, researchers have conducted numerous studies on rolling mill vibration monitoring and bearing fault diagnosis. However, the problem of rolling mill bearing faults has not been solved completely. With the rapid development of the strip mill and the use of new technologies, many complex forms and characteristics of roll-bearing faults in strip mills have emerged. Thus, it is necessary to develop new diagnostic approaches in order to address this problem. The contributions of this research are as follows: (1) A rolling mill vibration acquisition system is developed and designed, and the layout position of the acceleration sensor on the rolling bearing is discussed. (2) The basic theories of the fast spectral kurtosis method and PCA dimension reduction technique are described. (3) The SSA is employed to realize the intelligent, optimized, self-adaptive function of DBN. Furthermore, the firefly disturbance algorithm is used to improve SSA-DBN. Thus, an improved SSA-DBN method is obtained to ensure comprehensive diagnosis of faults. (4) According to the analysis of the data samples collected from the rolling mill fault experimental platform, the proposed method achieves better diagnostic performance than other methods. Finally, the optimal placement of sensors for rolling mill fault diagnosis is experimentally demonstrated.

## 2. Vibration Data Acquisition System of a Hot-Rolling Mill

**Figure 1:**
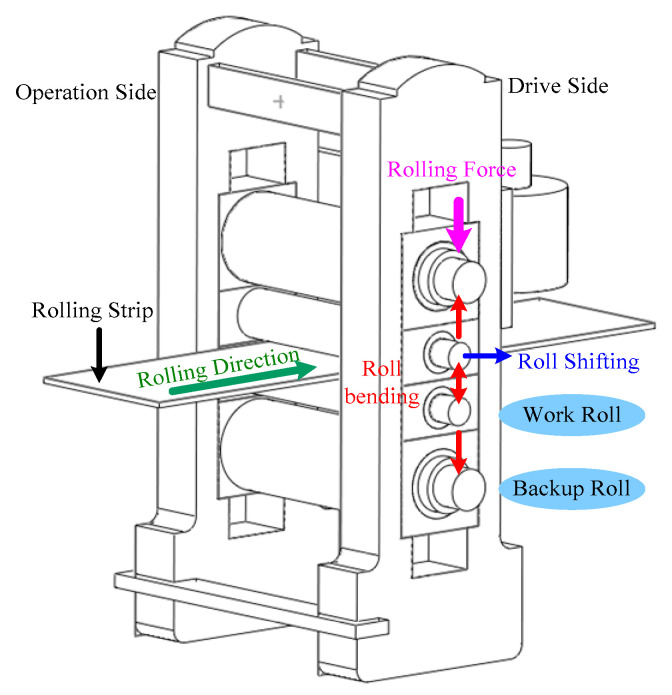
F2 mill housing structure of the 1780 mm hot strip-rolling mill.

The 1780 mm hot tandem mill unit of Chengde Iron and Steel Company (Chengde, China), consists of one roughing mill and five finishing mills. Among them, the finishing mills F1–F5 are five high-rolling mills that are arranged constantly, and the distance between adjacent stands is 6 m. It was found at the rolling site that F2 often vibrated and formed vibration marks on the strip surface, which reduced the surface accuracy of the strip. Therefore, we have designed and developed a system that monitors rolling mill vibration to collect its signals. Figure 1 shows a schematic of the F2 mill housing structure of the hot-rolling mill.

**Figure 2:**
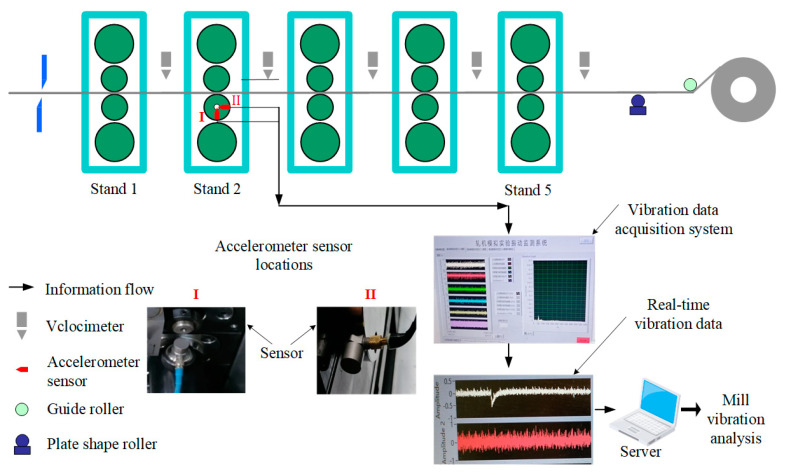
Vibration data acquisition system of the rolling mill.

Through field observation and monitoring activities, technicians found that the most obvious source of the F2 mill vibration was the location of the lower work roll drive side bearing. Therefore, two acceleration sensors (I and II) are arranged here. Sensor I is located at the axial end of the lower work roll bearing, while sensor II is located at the radial end. A field server is used to store and display the vibration signals that are extracted by the acceleration sensors instantaneously. Moreover, a production data monitoring system records all the rolling process data of the F2 mill housing during the whole test period. Thus, the conditions and the vibration conditions of the rolling process and the rolling mill at each specific moment can be obtained, respectively, as shown in Figure 2.

## 3. Signal Processing and Data Dimensionality Reduction

### 3.1. Fast Kurtogram

The commonly used time–frequency representation methods are classified into linear and nonlinear methods, both of which can map 1D time domain signals to 2D time-frequency planes in order to comprehensively reflect the time–frequency joint attributes of non-stationary signals [21,22]. Effective use of these methods can reveal the time and frequency performance of the energy contained in the rolling mill vibration signals. Some widely implemented time–frequency investigation methodologies for vibration signals include short-time Fourier transform (STFT), wavelet transform (WT), S transform, Hilbert–Huang transform (HHT), and Wigner–Ville distribution (WVD). These methods have their advantages and disadvantages. They must be used flexibly according to specific problems; if they are not handled properly, they may cause significant errors and yield unrealistic results.

The main drawback of STFT is that owing to the limitation of the uncertainty principle in terms of the time–frequency resolution, it is impossible to optimize both time and frequency concurrently. Moreover, the window mapping of STFT is constant and is not self-adaptable. Although the WT method overcomes some of the shortcomings of STFT, its division of the time–frequency plane is rather mechanical. There is no specific method for selecting the primary function, which must be determined repeatedly experimentally or through experience. The S-transform is a time–frequency analysis method developed by combining STFT and WT. Although it has many advantages, its basic wavelet function is fixed and has limited practical applications. The HHT method is suitable for non-linear and non-stationary signal analysis. However, it lacks a strictly theoretical basis. Moreover, it suffers from boundary effects and mode confusion in practical applications, and it can easily produce false frequency components. Although WVD compresses the cross-term interference of multi-component signals to a certain extent, some of its edge characteristics are severely damaged, which reduces its time–frequency focusing. Compared to the aforementioned methods, the fast spectral kurtosis method used in this study has obvious advantages; it allows self-adaptive selection of the resonant demodulation band parameters and does not require any set parameters. Hence, the model is easy to use. Therefore, fast spectral kurtosis is selected in the method of rolling mill vibration signal extraction to reduce the number of parameters that must be set.

Some researchers have imposed four constraints on the kurtosis analysis method to increase the generalizability of the signal conversion procedure. This makes the model more sensitive to signals having non-stationarity characteristics. The elaborate process of the fast kurtosis approach has been described in Ref. [23]. It is denoted by

(1) $$K\left(f\right)=\frac{\langle S{\left|t,f\right|}^{4}\rangle }{{\langle S{\left|t,f\right|}^{2}\rangle }^{2}}-2$$

where *f* ≠ 0, *S*(*t*, *f*) represents the complex envelope of vibration signal *x*(*t*) at frequency *f* and 〈.〉 denotes the computation for the average of the time [24]. Furthermore, *S*(*t*, *f*) can be computed by

(2) $$S\left(t,f\right)=\int_{-\infty }^{+\infty }x\left(t\right)w\left(t-\tau \right)e^{-2\pi ft}dt$$

where *w*(*t*) represents the window mapping used in this method.

The fast spectral kurtosis method can effectively process the original vibration signals of the rolling mill bearings, convert the rolling mill vibration time domain signals into 2D time–frequency images, improve signal recognition, and facilitate the characteristic recognition of different vibration states of the rolling mill bearings.

### 3.2. PCA Dimension Reduction Theory

The original vibration signal of the rolling mill bearings recorded by the vibration data acquisition system suffers from high data feature dimension and is difficult to process. Therefore, PCA is adopted to decrease the number of dimensions in the original vibration fault signal of the hot rolling mill in order to achieve rapid data processing [25,26]. The detailed steps are as follows.

The acquisition system is set up to record *m* pieces of *n*-dimensional original rolling mill vibration signal data.

Step 1: Form the original rolling mill vibration signal into matrix $X=\left\{x_{i,j}:x_{i,j}\in R^{n\times m}\right\}$, where each row of vector $x_{i}\in R^{1\times m}$, *i* = 1, …, *n*, represents a measure. Further, each column vector $x_{j}\in R^{n\times 1}$, *j* = 1, …, *m*, denotes a sample. In addition, calculate the mean of *x_j_*, defined by

(3) $${\bar{x}}_{j}=\frac{1}{m}\sum_{j=1}^{m}x_{j}$$

Step 2: Subtract the average value of each dimensional feature *x_j_*, i.e., standardize the data. Then, calculate the covariance matrix *C*, which is a symmetric matrix; it is expressed as follows:

(4) $$C=\frac{1}{m-1}\sum_{j=1}^{m}\left(x_{j}-{\bar{x}}_{j}\right){\left(x_{j}-{\bar{x}}_{j}\right)}^{T}$$

Then, calculate the eigenvalues *λ_i_*, *i* = 1, …, *n* of matrix *C* and the corresponding eigenvectors *v_i_*, *i* = 1, …, *n*.

Step 3: Sort the eigenvalues in descending order and select the maximum *Z*. Subsequently, the cumulative contribution ratio of the first *Z* principal components can be calculated as *α*, which is denoted by

(5) $$\alpha =\sum_{i=1}^{z}\lambda_{i}/\sum_{i=1}^{n}\lambda_{i}$$

Step 4: A cumulative contribution rate of *α* ≥ 0.85 can ensure minimum loss of the original rolling mill vibration data. At this time, the previous *Z* eigenvectors can be formed into a new matrix, and the data can be transformed into the space of matrix *P*, where $P=\left(v_{1},v_{2}\cdots ,v_{z}\right)$. Then, the data matrix reduced to *Z* dimensions can be obtained.

(6) $$X^{\prime }=PX$$

Thus, a new dataset of rolling mill-bearing vibration signals is generated. Compared to the original vibration signal, the new signal dataset has a lower dimension, retains the most important data features, consumes less time, and reduces the computational cost significantly.

## 4. Proposed Model

### 4.1. Sparrow Search Algorithm (SSA)

Intelligent optimization algorithms constitute a type of random search algorithm inspired by biological swarm intelligence or physical phenomena. Several conventional optimization methodologies, such as particle swarm optimization, grey wolf optimizer, and genetic algorithm, have been widely implemented. These methods are used to optimize the super-parameters of neural networks because of their simplicity, flexibility, and efficiency. In 2020, Xue and Shen [27] proposed the SSA, a new optimization method. This algorithm is principally inspired by sparrows’ foraging behavior. It outperforms all the aforementioned methods in terms of accuracy, convergence speed, stability, and robustness.

The algorithm has three main components: producers, scroungers, and vigilantes. The producers are mainly responsible for searching an area with a large amount of food and supplying the foraging area and environment for the scroungers. As long as better food sources can be found, every sparrow could become a producer, which means that the identities of producers and scroungers change dynamically; however, their ratio in the entire population remains the same. The sparrows (vigilantes) at the edge of the group will send an alarm signal once they encounter a predator. When the alert level is higher than the safety level, the sparrow at the edge of the group will move toward the inside of the group and find a safer position.

The sparrows find the optimal parameters by calculating the fitness function to constantly update their position. The sparrow position matrix is expressed as follows:

(7) $$X=\left[\begin{array}{ccccc} x_{1,1} & x_{1,2} & \cdots & \cdots & x_{1,d}\\ x_{2,1} & x_{2,2} & \cdots & \cdots & x_{2,d}\\ \vdots & \vdots & \vdots & \vdots & \vdots \\ x_{n,1} & x_{n,2} & \cdots & \cdots & x_{n,d} \end{array}\right]$$

where *d* and *n* represent the number of variables and observations (sparrows) to be optimized, respectively. The function that measures the fitness value is denoted by

(8) $$F_{X}=\left[\begin{array}{ccccc} f\left(\left[x_{1,1}\right.\right. & x_{1,2} & \cdots & \cdots & \left.\left.x_{1,d}\right]\right)\\ f\left(\left[x_{2,1}\right.\right. & x_{2,2} & \cdots & \cdots & \left.\left.x_{2,d}\right]\right)\\ \vdots & \vdots & \vdots & \vdots & \vdots \\ f\left(\left[x_{n,1}\right.\right. & x_{n,2} & \cdots & \cdots & \left.\left.x_{n,d}\right]\right) \end{array}\right]$$

where *f*(.) represents the individual’s fitness number.

According to Equations (7) and (8), the updated location for the producers is denoted by

(9) $$X_{i,j}^{t+1}=\left\{\begin{array}{l} X_{i,j}^{t}\cdot \exp\left(-\frac{i}{\delta \cdot iter_{\max}}\right)\;\;if\;R_{2}<ST\\ X_{i,j}^{t}+Q\cdot L\;\;\;\;\;if\;R_{2}\geq ST \end{array}\right.$$

where *X_i_*_,_*_j_* denotes the *i*th sparrow in the *j*th dimension, *t* denotes the iteration numbers, *j* = 1, 2, 3,…, *d*, *iter_max_* is a constant representing the maximum number of iterations, $\delta \in \left[0,1\right]$ denotes a randomly generated number, *R*_2_ and *ST* denote the warning and safety values, respectively, *Q* is a randomly generated number with a normal distribution, and *L* equals 1 with all elements being a 1 × *d* matrix.

(10) $$X_{i,j}^{t+1}=\left\{\begin{array}{l} Q\cdot \exp\left(-\frac{X_{worst}^{t}-X_{i,j}^{t}}{i^{2}}\right)\;\;\;\;\;\;\;\;\;if\;i>\frac{n}{2}\\ X_{P}^{t+1}+\left|X_{i,j}^{t}-X_{P}^{t+1}\right|\cdot A^{T}{\left(AA^{T}\right)}^{-1}\cdot L\;otherwise \end{array}\right.$$

where *X_P_* denotes the best position occupied by the current producers and *X_worst_* denotes the current global worst position. Further, *A* denotes a 1 × *d* matrix, and *A^T^* is the transposed determinant of *A*. Whenever i>n/2, the *i*th follower having a low fitness number cannot obtain food. At this time, it must fly elsewhere to feed.

When danger is detected, the location update of the vigilantes is expressed as follows:

(11) $$X_{i,j}^{t+1}=\left\{\begin{array}{l} X_{best}^{t}+\beta \cdot \left|X_{i,j}^{t}-X_{worst}^{t}\right|\;\;\;\;if\;f_{i}>f_{g}\\ X_{i,j}^{t}+K\cdot \left(\frac{\left|X_{i,j}^{t}-X_{worst}^{t}\right|}{\left(f_{i}-f_{w}\right)+\varepsilon }\right)\;\;if\;f_{i}=f_{g} \end{array}\right.$$

where *X_best_* denotes the current global optimal location; *β* and *K* are random numbers having normal distributions with an average of 0 and variance of 1 and are also control parameters of the step size; *f_i_* denotes the fitness value of the current individual sparrow; *f_g_* and *f_worst_* represent the current global optimal and worst fitness values, respectively; and *ε* represents a constant used to avoid the zero denominator case. Furthermore, *f_i_* > *f_g_* indicates that the sparrows at the edge of the entire population are sensitive to predators, whereas *f_i_* = *f_g_* denotes that the sparrows in the center of the entire population are aware of the danger and must move closer to the other members of the population to avoid being potential prey.

### 4.2. Deep Belief Network (DBN)

**Figure 3:**
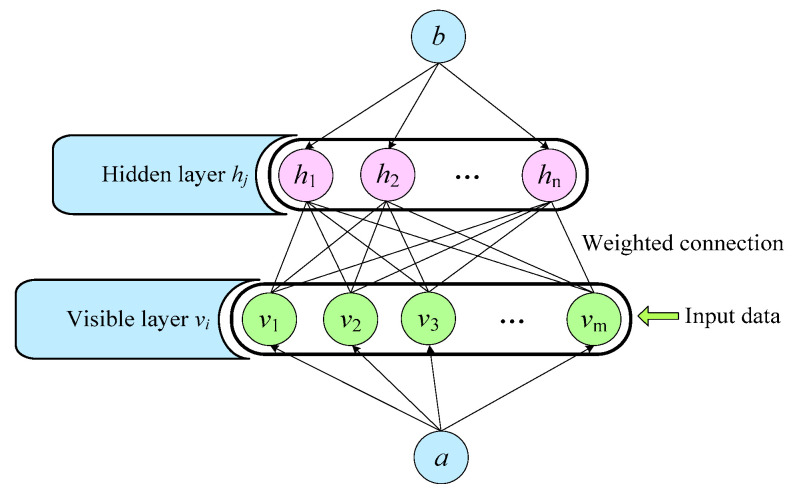
Structure of the restricted Boltzmann machine.

The restricted Boltzmann machine is a random neural network composed of visible and hidden layers. Independent neurons exist in the same layer, while dependent neurons are connected in different layers, as shown in Figure 3, where *m* and *n* are the nodes of the visual and hidden layers, respectively, and *v_i_* and *h_j_* are the input of the visual layer and the output of the hidden layer, respectively.

The energy mapping for the RBM is defined by

(12) $$E\left(v,h\left|\theta \right.\right)=-\sum_{i=1}^{m}\sum_{j=1}^{n}h_{j}w_{ij}v_{i}-\sum_{i=1}^{m}a_{i}v_{i}-\sum_{j=1}^{n}b_{j}h_{j}$$

where $\theta =\left\{\left\{w_{ij}\right\},a,b\right\}$ represents the vector parameter of the RBM, *w_ij_* denotes the weighted relationship between the nodes of the visible and hidden layers and *a_i_* and *b_j_* denote the coefficient of bias for the visible and hidden layers, respectively.

The RBM has the joint probability distribution defined in Ref. [28] by

(13) $$p\left(v,h\left|\theta \right.\right)=\frac{1}{Z\left(\theta \right)}e^{-E\left(v,h\left|\theta \right.\right)}$$

where *Z*(*θ*) denotes the normalization term expressed by

(14) $$Z\left(\theta \right)=\sum_{v,h}e^{-E\left(v,h\left|\theta \right.\right)}$$

The visible and hidden layers have conditional probability distributions defined by

(15) $$p\left(h_{j}=1\left|v\right.\right)=\sigma_{s}\left(b_{j}+\sum_{i=1}^{m}w_{ij}v_{i}\right)$$

(16) $$p\left(v_{i}=1\left|h\right.\right)=\sigma_{s}\left(a_{i}+\sum_{j=1}^{n}w_{ij}h_{j}\right)$$

where the activation mapping is denoted by *θ*, and the vector parameters above can be obtained as the optimal parameters through the maximum likelihood function. The formula is expressed by

(17) $$\hat{\theta }=\arg\max\ln P\left(\theta \left|x_{1},x_{2},\cdots ,x_{k}\right.\right)=\frac{1}{k}\sum_{i=1}^{k}\ln P\left(x_{i}\left|\theta \right.\right)$$

where the number of training data is represented by *k.* To prevent premature convergence of the algorithm or instability after multiple iterations, Professor Hinton proposed the contrast divergence (CD) algorithm [29], which can accelerate the calculation and further obtain the estimated parameters. The update process of parameters $\theta =\left\{\left\{w_{ij}\right\},a,b\right\}$ is expressed by

(18) $$\begin{array}{l} \Delta w_{ij}=\eta \left({\langle v_{i}h_{j}\rangle }_{data}-{\langle v_{i}h_{j}\rangle }_{recon}\right)\\ \Delta a_{i}=\eta \left({\langle v_{i}\rangle }_{data}-{\langle v_{i}\rangle }_{recon}\right)\\ \Delta b_{j}=\eta \left({\langle h_{j}\rangle }_{data}-{\langle h_{j}\rangle }_{recon}\right) \end{array}$$

where $\eta \in \left(0,1\right)$ represents the learning rate, 〈.〉*_data_* denotes the expected value based on the defined distribution of the training dataset, and 〈.〉*_recon_* denotes the expected value based on the defined distribution of the reconstructed deep belief network model. When *k* = 1, the contrast divergence algorithm has an ideal effect; hence, the form of the CD-1 method is generally employed to obtain the best parameters.

**Figure 4:**
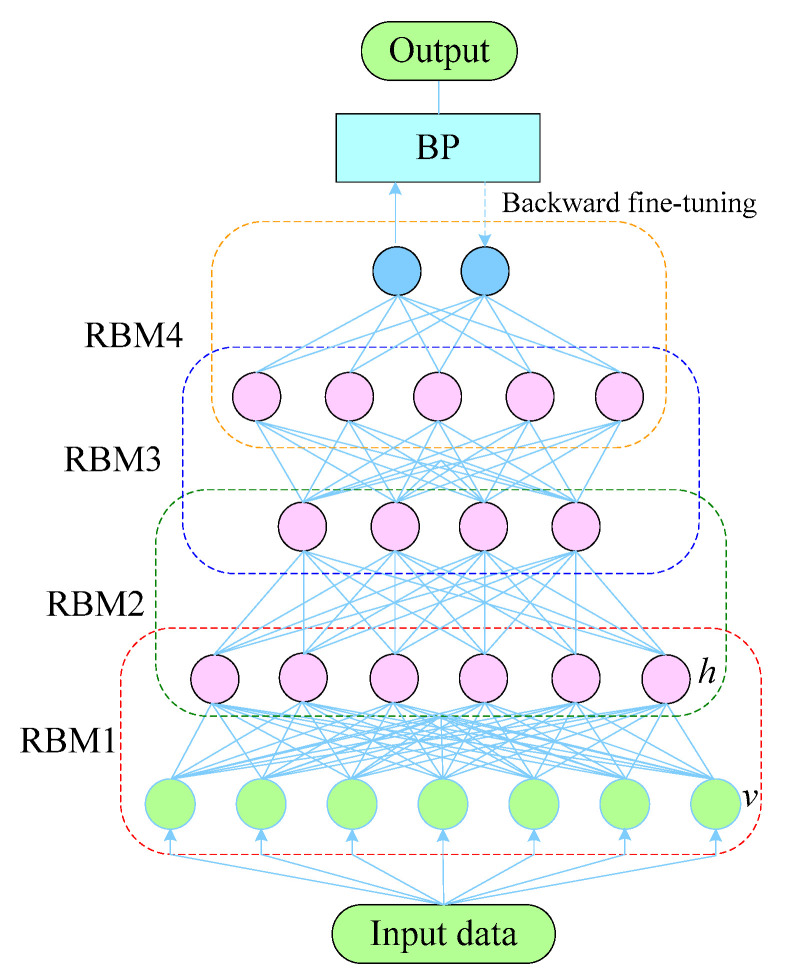
Fundamental skeleton of DBN.

The existing literature shows that the representation ability of a single RBM for complex raw data is often insufficient. Hence, multiple RBMs are generally stacked into a deep confidence network to extract deep-seated features one layer at a time. The basic structure of DBN is presented in Figure 4. As can be seen, the first and second layers (visible and hidden layers) constitute the first RBM, namely RBM1, and the second and third layers constitute the second RBM, namely RBM2. The construction process continues in this manner. Thus, the stacking forms multiple RBMs. Multiple RBMs can obtain essential features by using the original vibration signals; however, they cannot directly cluster the data. Therefore, a back propagation (BP) layer should be added to the top of the stacked RBM for reverse fine-tuning to obtain a final model of the DBN.

Figure 4 shows that the training procedure of DBN consists of two processes: forward unsupervised pre-training and backward supervised fine-tuning. In the forward training stage of DBN, the greedy unsupervised learning mechanism is employed for bottom-to-top transfer, and the feature extraction of the rolling mill bearing vibration data is finally completed. After the unsupervised training, the BP algorithm is employed. The objective of back-propagation is to minimize the residual between the reconstructed classification outcomes and the real observations. The super-parameter $\theta =\left\{w,a,b\right\}$ of the whole network is fine-tuned to achieve the optimal solution.

### 4.3. Deep Belief Network Based on Improved Sparrow Search Algorithm (ISSA-DBN)

**Figure 5:**
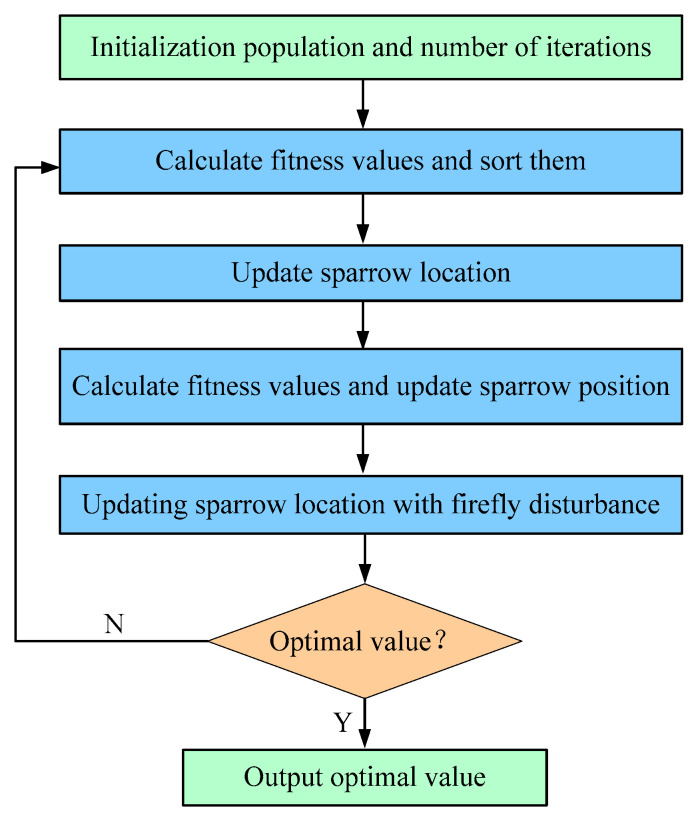
Improved sparrow search algorithm (ISSA) process.

Producers, scroungers, and vigilantes are prone not only to population aggregation and falling into local optima but also to reduced population diversity in the SSA. Therefore, after running the sparrow search, this study uses the firefly algorithm to disturb the optimization of all the sparrows, and if a better result is found, the sparrow position is updated. Firefly disturbance is a global intelligent optimization method that simulates the flashing behavior of fireflies [30]. The improved sparrow search algorithm (ISSA) improves not only the diversity of the population location transformation but also the spatial search and robustness of the sparrow optimization algorithm. The specific step is to add firefly disturbance after the sparrow population location is updated. The disturbed sparrow location is expressed as follows:

(19) $${\left(X_{i,j}^{t+1}\right)}^{*}=X_{i,j}^{t+1}+\beta_{0}e^{-\gamma r^{2}}+\alpha \varepsilon$$

where *r* represents the distance between the same sparrow before and after the disturbance, $\beta_{0}e^{-\gamma r^{2}}$ is the attraction, *β*_0_ represents the maximum attraction when the disturbance distance is zero, *γ* is the attraction attenuation parameter, $\gamma \in \left[0,+\left.\infty \right)\right.$, and *αε* is a random item. The flow of the sparrow optimization algorithm based on firefly disturbance is shown in Figure 5.

**Figure 6:**
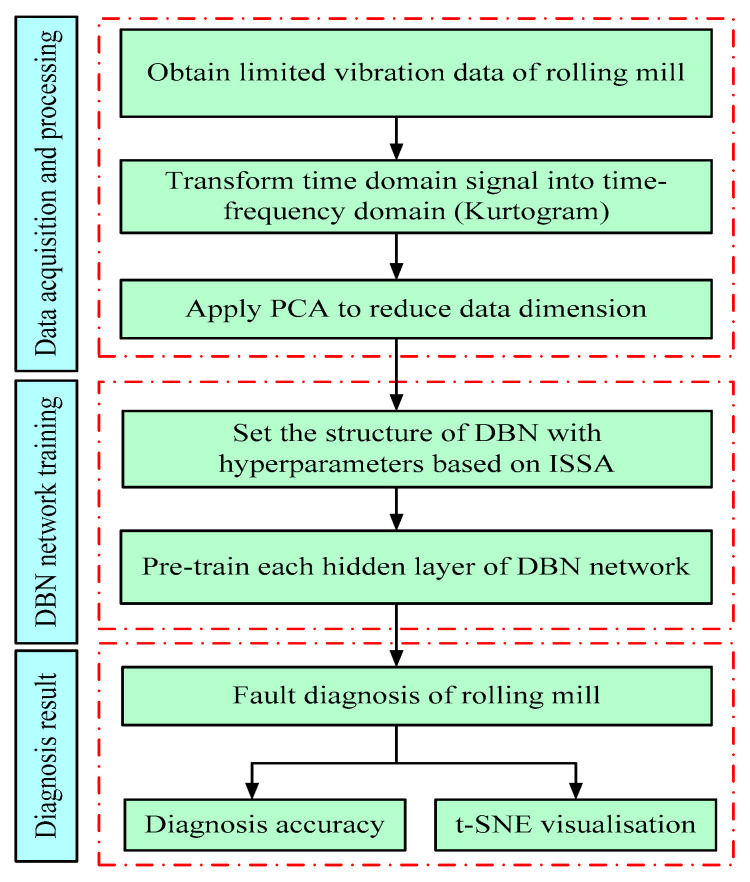
Flowchart of fault diagnosis of rolling mill using ISSA-DBN.

In the process of rolling mill vibration fault diagnosis, the diagnostic performance of DBN plays a critical role. In particular, it affects the outcomes of the data classification or prediction. As is well known, the performance of a DBN mainly depends on its structure and the setting of various parameters; the number of neurons in the hidden layer is a significant parameter in the network structure. Too many or too few neurons will reduce the generalization ability and the fitting effect of a neural network, and the feature extraction will not be effective. Therefore, selecting the optimal number of hidden layer neurons is important for detecting the health state of the rolling mill. The general method is based on expert experience; however, this will lead to significant overhead and randomness in the network performance. Therefore, this study adjusts the number of neurons available in the hidden layer of the DBN using the improved SSA (ISSA). Thus, the DBN can rapidly find the best structure to realize the intelligent optimization self-adaptive function of the neural network, so that the rolling mill bearing fault can be accurately diagnosed in the case of limited data samples. The rolling mill fault diagnosis process based on ISSA-DBN is shown in Figure 6.

## 5. Arrangement of Rolling Mill Experimental Platform and Sensors

### 5.1. Experimental Platform and Data Acquisition of Rolling Mill Faults

**Figure 7:**
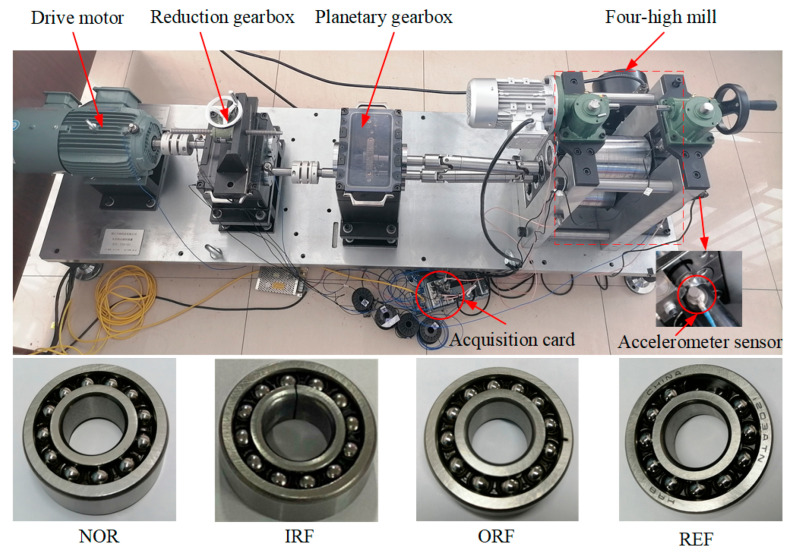
Rolling mill fault diagnosis test platform and fault bearing type.

Figure 7 shows the rolling mill fault diagnosis test platform and fault bearing types. The platform is scaled equally and used for the experiment according to the actual 1780 mm hot rolling mill; hence, it is the same as the fault representation in Figure 2. The rolling mill fault diagnosis test platform mainly consists of the drive motor, coupling, reduction gearbox, gear base, four high rolling mills, and related control parts. The lower-work roll bearing can be replaced freely, which facilitates the replacement of different types of faulty bearings. The two sensors installed at the axial and radial ends of the lower-work roll bearing seat can collect the bearing vibration signals. According to the different experimental scenarios designed, the collected bearing vibration data are marked as follows: normal (NOR), inner ring fault (IRF), outer ring fault (ORF), and rolling element fault (REF).

The original vibration signals of the rolling mill are collected at three distinct rolling speeds of 600 rpm, 900 rpm, and 1200 rpm, and the sampling rate is 10,240 Hz. The collected data are cut and segmented to form training and test datasets. Each fault type in the training dataset A/B/C/D contains 20/40/60/80 training samples, respectively, and the number of data points in the test dataset is 100. The specific sample allocation strategy is shown in Table 1.

**Figure 8:**
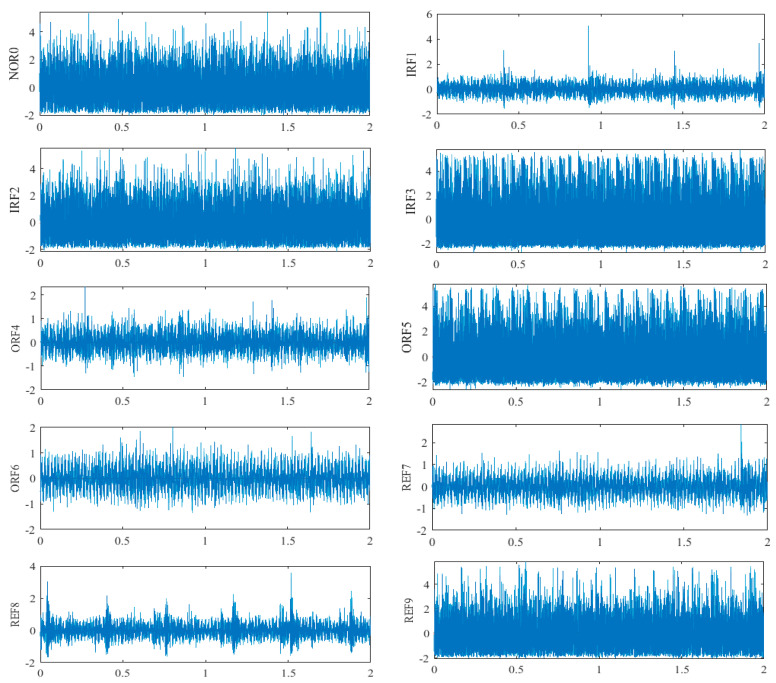
Time domain waveform of different fault signals of rolling mill bearings collected by sensor I.

**Figure 9:**
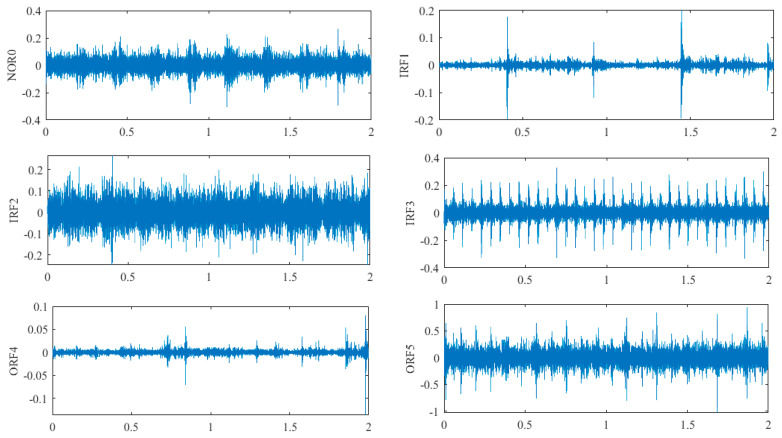

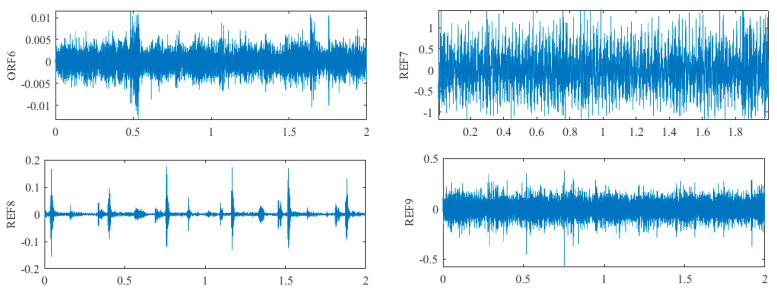
Time domain waveform of different fault signals of rolling mill bearings collected by sensor II.

When the rolling mill rolls the strip steel, a defective bearing will cause a series of vibrations in the work roll, and the acceleration sensor installed on the roll bearing pedestal will receive a series of vibration signals. The time domain waveforms of the signals collected by the axial and radial end sensors of the bearing pedestal under 10 fault vibration states are shown in Figure 8 and Figure 9. Starting from the time domain waveform, although preliminary fault identification can be conducted, there are still some faults that are difficult to distinguish, such as NOR0, IRF2, IRF3, ORF5, and REF9 in Figure 8 and IRF2, ORF5, ORF6, and REF9 in Figure 9. Therefore, better methods should be adopted for feature extraction and fault diagnosis of the vibration signals.

**Figure 10:**
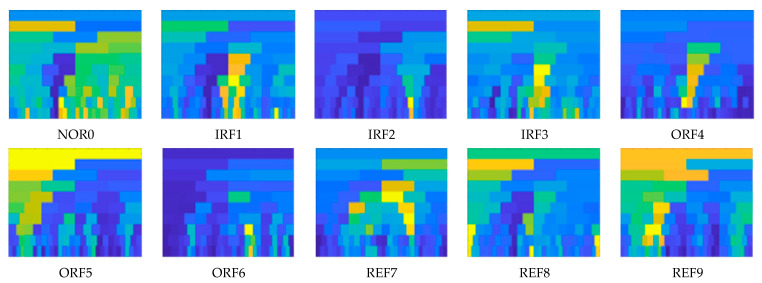
Two-dimensional spectral kurtosis of different fault signals of rolling mill bearings collected by sensor I.

**Figure 11:**
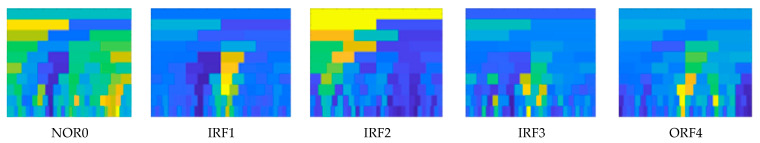

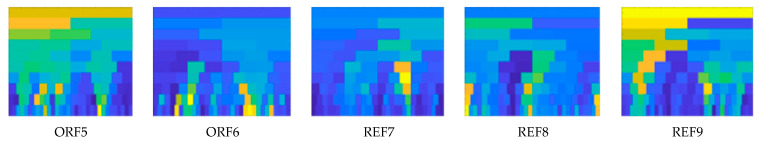
Two-dimensional spectral kurtosis of different fault signals of rolling mill bearings collected by sensor II.

Figure 10 and Figure 11 show 2D spectral kurtosis diagrams of different fault signals of the rolling mill collected by sensor I and sensor II of the rolling mill work roll bearing, respectively. Spectral kurtosis is highly sensitive to the transient impact in the rolling mill bearing fault signal. Thus, it can effectively identify and determine the frequency band position and interval of the fault signal and has a strong fault feature extraction ability. Compared with the time domain waveform, the spectral kurtosis can display the time–frequency information of different bearing fault signals through the chromatic graph grid, and there is no case in which it is difficult to distinguish the time-domain signal. However, 2D spectral kurtosis cannot easily determine the fault type to which a time–frequency signal belongs; hence, it is necessary to implement the proposed ISSA-DBN approach to determine the bearing fault of the rolling mill.

### 5.2. Selection of Optimal Diagnosis Position of the Sensor

**Figure 12:**
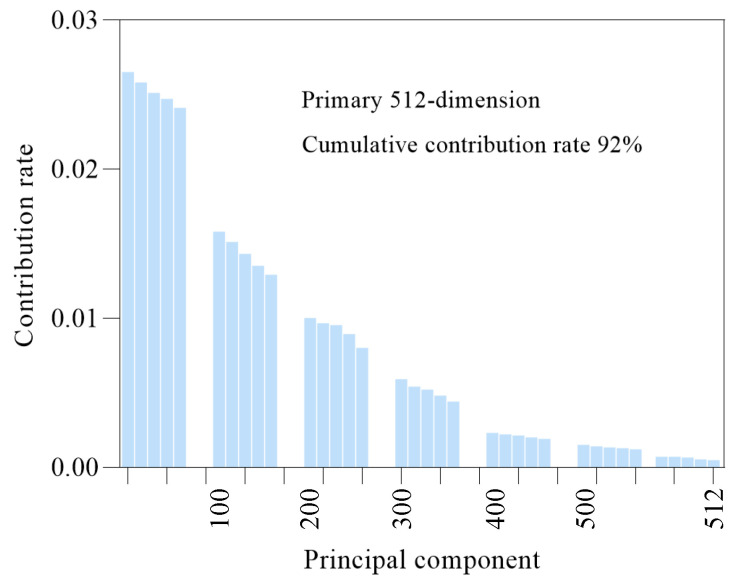
Contribution rate of data characteristics in sensor I.

**Figure 13:**
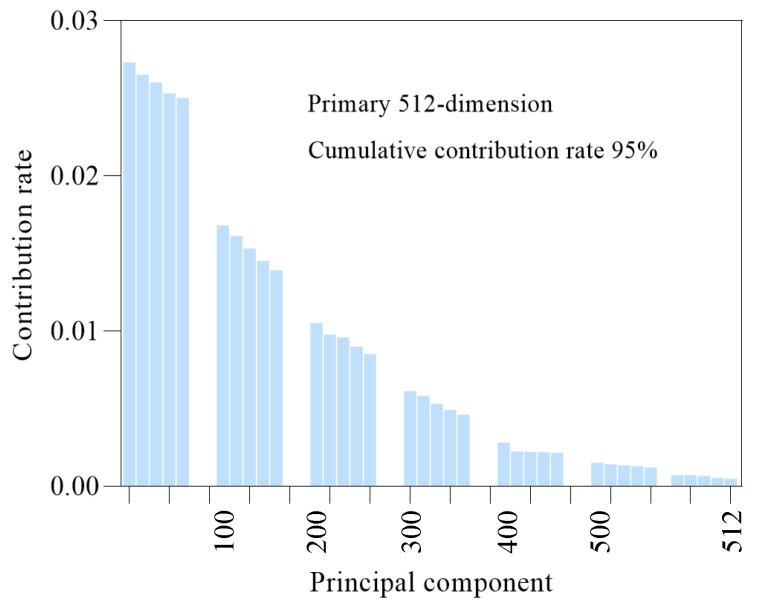
Contribution rate of data characteristics in sensor II.

As the characteristic dimension of the 2D spectral kurtosis diagram is set to be 32 × 32 × 3, expanding it into 1D data and inputting it into the ISSA-DBN method proposed in this study may cause dimension explosion and gradient disappearance when the gradient decreases. To better retain the original vibration signal characteristics of the rolling mill bearings and reduce the computational burden, the PCA method is used to decrease the number of dimensions. Figure 12 and Figure 13 show the contribution rate curves cumulatively. After the PCA technology is adopted, the cumulative contribution rate of the first 512 dimensional principal components exceeds 92%; the important features of the 2D spectral kurtosis image are retained. Therefore, the first 512 dimensional features are input into the ISSA-DBN method for fault classification.

In the experiment, the learning rate of this method is 0.001, and each experiment is repeated 10 times to reduce the influence of randomness. All the experiments are carried out using MATLAB on an I5-7500 CPU with 4 GB RAM. After intelligent optimization self-adaptation, the final number of DBN layers is five, i.e., input, output, and three hidden layers. The number of input layer nodes is set to 512, and the number of output layer nodes is set according to the fault type, i.e., 10. Therefore, the optimal structure of DBN is 512-390-251-89-10. Table 2 presents the comparison results of the proposed method and other available methods in terms of the average accuracy of rolling mill bearing fault classification.

The accuracy of fault diagnosis using the proposed ISSA-DBN method is higher than that of the other methods, regardless of whether the data collected by sensor I or sensor II are considered. Moreover, the data collected by sensor II have higher accuracy in terms of the fault classification rate compared to sensor I, i.e., the data collected by the sensor attached at the radial end of the work roll bearing are better than the data collected by the sensor attached at the axial end. Further observation shows that in dataset D, i.e., when the number of samples is large, the data collected by sensor II are used for the experiments, and the accuracy of the four-fault diagnosis methods is higher than 91%. However, in the more challenging small sample dataset A, higher classification accuracy is achieved for the ISSA-DBN, which is 17.6% higher than that of the deep neural network (DNN) with the worst performance. Compared with the DBN before improvement, the diagnostic performance of ISSA-DBN is 6.7% higher and 4.6% higher than that of the convolutional neural network (CNN) with the best performance. Therefore, the following conclusions can be drawn: (1) This method achieves excellent results for all the sensors, with the highest accuracy and the lowest deviation. (2) The accuracy of all the methods with sensor II is higher than that with sensor I. (3) In particular, as the task becomes more severe, the accuracy of this method becomes significantly lower than that of the other methods, indicating that it is more suitable for small samples.

**Figure 14:**
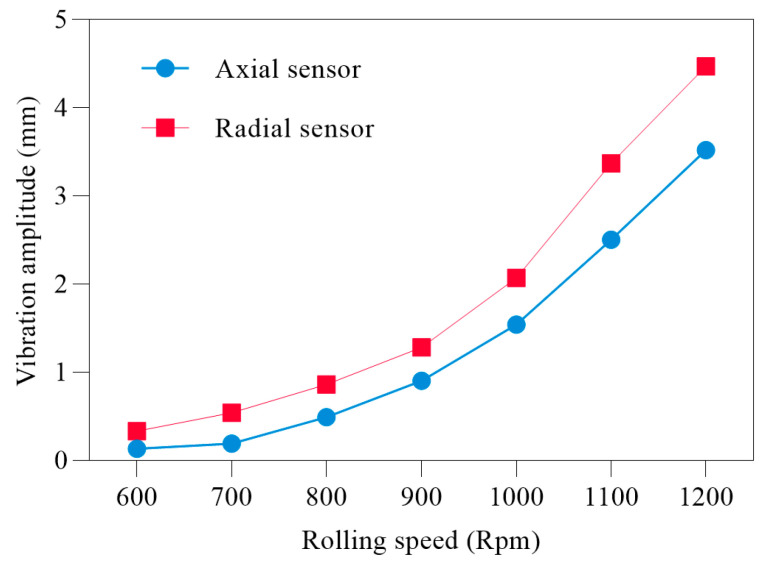
Influence of rolling speed on sensor amplitude.

To better reflect the superiority of the data collected by sensor II, further verification is conducted from the perspective of rolling speed change. Figure 14 shows the influence of different rolling speeds on the sensor amplitude. As can be seen, when the rolling speed increases, the amplitude of both the sensors increases; however, sensor II is more sensitive to vibration, indicating that the data it collects contain more useful information. This is because the radial end of the bearing is affected by the rolling force from the vertical direction of the rolling mill as well as by many vibration parameters and process parameters between the rolling mill roll systems. Therefore, the data collected by sensor II of the rolling mill bearing are used to compare the fault classification accuracy in different situations.

## 6. Case Analysis and Discussion

**Figure 15:**
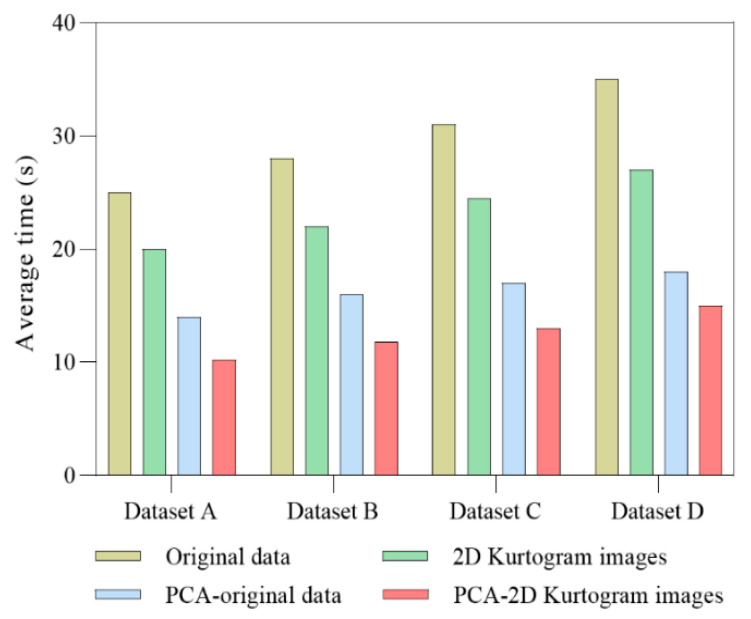
Average number of training epochs of different data processing methods.

Under the same computer configuration environment, the dataset A/B/C/D is input into ISSA-DBN using different processing methods, and the average epochs required on the training dataset can be recorded, as shown in Figure 15.

As can be seen, for different data samples, the original vibration data require more training epochs, and as the number of samples increases, the number of epochs necessary also increases. Moreover, the number of epochs required on the training dataset for 2D spectral kurtosis image information is smaller than that for the original signal. After using PCA technology to reduce the dimension, the number of training epochs of different datasets decreases rapidly owing to the data dimension reduction. Among them, the PCA-2D kurtogram images require the fewest epochs, which shows that the dimensionality reduction method used in this study can accelerate the training procedure and enhance the calculation efficiency significantly.

**Figure 16:**
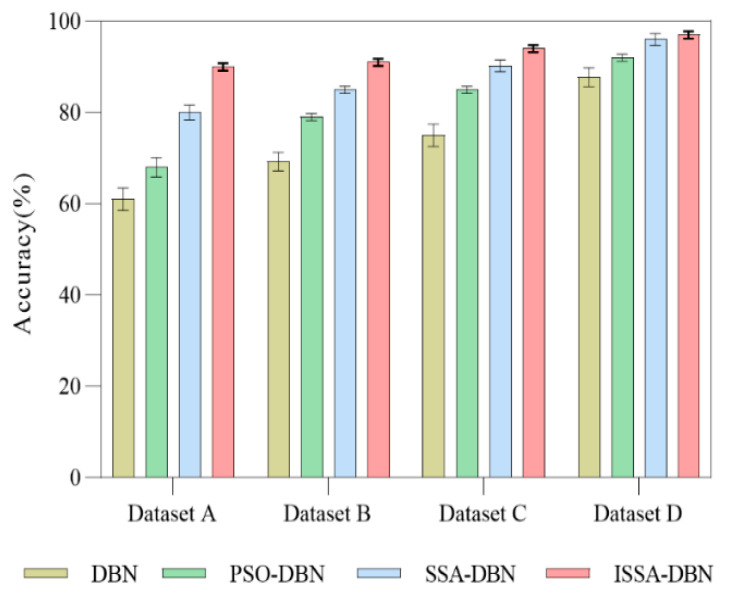
Classification performance of different diagnostic methods.

Figure 16 compares the fault diagnosis performance of different methods. As can be seen, in the small sample dataset A, the classification accuracies of DBN, PSO-DBN, and SSA-DBN are 61.1%, 68.2%, and 80.6%, respectively, and the classification accuracy of ISSA-DBN is 92.4%. As the data samples increase in dataset D, the fault diagnosis performance of different methods improves considerably. The accuracy of DBN is 87.8%, and that of PSO-DBN is 92%. The diagnosis and classification results of SSA-DBN before and after improvement are the same, i.e., both are above 96%. Moreover, the error of the proposed approach is small, which indicates that the method has a more stable training process, which further shows that ISSA-DBN has more advantages in the classification of rolling mill bearing faults under small sample data.

**Figure 17:**
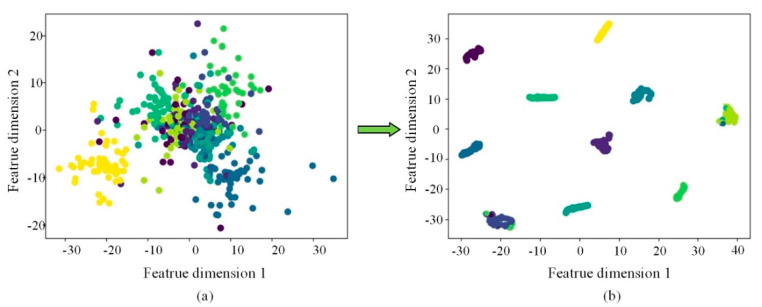
Visual analysis of characteristics of small sample data. (**a**) the visualization effect of the original test sample. (**b**) the visualization effect of the ISSA-DBN method.

To further demonstrate the classification performance of the proposed approach, the t-distributed stochastic neighbor embedding (t-SNE) methodology was adopted to visually examine the features of a small sample dataset A, as shown in Figure 17. Figure 17a shows the original test sample. As can be seen, almost all the data points are doped and overlapped together. The visualization effect of t-SNE after using ISSA-DBN to extract the data features is shown in Figure 17b. The proposed method can separate the mixed fault data and gather similar features. Although there is a small amount of overlap, the classification effect is relatively good overall.

**Figure 18:**
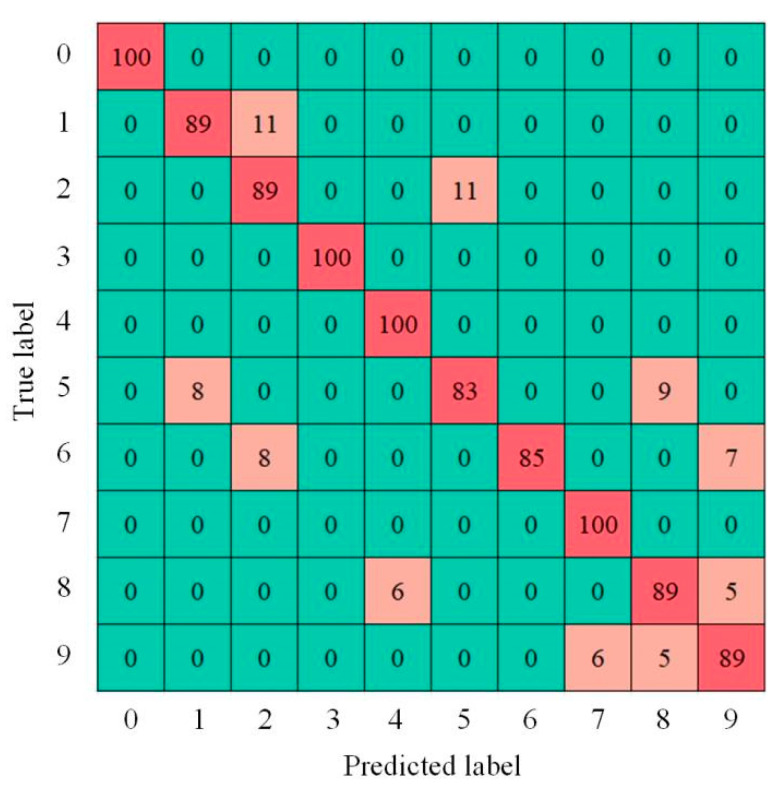
Confusion matrix of rolling mill bearing fault diagnosis results.

To demonstrate the classification of various samples more intuitively, Figure 18 shows the confusion matrix of the diagnosis results using the test data of a few samples of rolling mill bearing faults. The ordinate represents the real label, and the abscissa represents the forecast label. As can be seen, labels 0, 3, 4, and 7 have the highest accuracy, while label 5 has the lowest accuracy.

**Figure 19:**
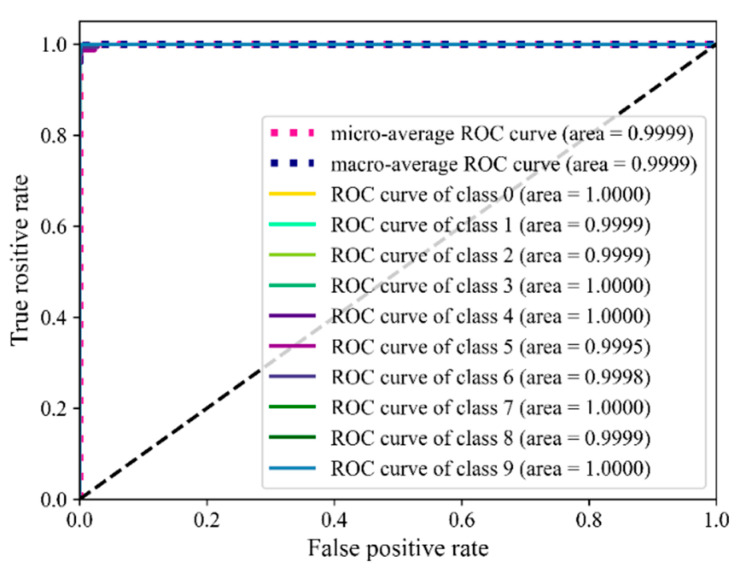
ROC curve of rolling mill bearing diagnosis results.

Figure 19 shows the outcomes of hot-rolling bearing fault diagnosis using the receiver operating characteristic (ROC) curve of the proposed approach as well as the diagnosis results of hot-rolling mill bearings. As can be seen, there are 10 categories. The area under the curve (AUC) of categories 0, 3, 4, 7, and 9 reaches 100%, and the AUC of the remaining categories is 99.95% or more. The AUC of both macro- and micro-ROC curves is 99.99%, indicating that ISSA-DBN has the characteristics of high sensitivity and low error rate and has a good diagnostic effect for rolling mill bearings.

## 7. Conclusions

This study proposed a method that diagnoses the faults of rolling mill bearings using limited data samples, namely the ISSA-DBN. The rolling production process was used to illustrate the proposed approach. The key contributions are as follows:

(1) The 2D spectral kurtosis image obtained using the fast spectral kurtosis method was shown to have richer data characteristics compared with the images generated by the original vibration signals of the rolling mill bearing. To further improve its diagnostic efficiency, the PCA method was employed to decrease the data dimension, which can not only prevent overfitting but also ensure good diagnostic performance of the network.
(2) The SSA algorithm can realize the intelligent optimization self-adaptive effect of DBN and achieve the best network structure configuration to enhance the generalization ability and classification accuracy of the network. Moreover, using the firefly disturbance algorithm to improve SSA can improve the spatial search ability and robustness of ISSA-DBN. Thus, the ISSA-DBN method was finally obtained to realize true fault diagnosis.
(3) A comparison of the fault classification accuracy of multiple diagnosis methods and the amplitude changes of sensors at different speeds showed that the proposed method achieves optimal performance at both sensor positions. Moreover, through experimental phenomena, it was found that the sensor installed at the radial end of the rolling mill bearing contains more effective information than the sensor installed at the axial end. Thus, this study provided empirical guidance for finding the best sensor position on the rolling mill bearing.
(4) Finally, the computational efficiency of the proposed method under different data processing methods and the classification performance with different diagnosis methods were discussed, which further proved that the proposed method has high accuracy and effectiveness in rolling mill bearing fault diagnosis with limited collected samples.

Future research will focus on the multi-source sensor fusion method that can be used to diagnose the faults of rolling mill bearings, roll vibration marks, and gearbox gears more accurately in order to ensure healthy operation of the rolling mills.

## Figures and Tables

**Table 1:** Description of experimental datasets.

Condition	Rpm	Label	Training Samples of Sensor I/II				Test Data
			The Dataset A	The Dataset B	The Dataset C	The Dataset D	
NOR	600	0	20	40	60	80	100
IRF	600	1	20	40	60	80	100
	900	2	20	40	60	80	100
	1200	3	20	40	60	80	100
ORF	600	4	20	40	60	80	100
	900	5	20	40	60	80	100
	1200	6	20	40	60	80	100
REF	600	7	20	40	60	80	100
	900	8	20	40	60	80	100
	1200	9	20	40	60	80	100
Total			200	400	600	800	1000

**Table 2:** Average accuracy comparison.

Methods (%)	The Dataset A		The Dataset B		The Dataset C		The Dataset D	
	Sensor 1	Sensor 2	Sensor 1	Sensor 2	Sensor 1	Sensor 2	Sensor 1	Sensor 2
DNN	73.3	74.8	77.9	78.6	79.1	83.2	86.5	91.3
SAE	81.1	82.9	84.2	84.9	85.9	89.7	89.7	93.4
DBN	83.6	85.7	86.4	88.1	88.7	89.3	91.7	93.5
CNN	85.3	87.8	89.5	91.8	91.7	93.0	93.5	94.1
ISSA-DBN	90.1	92.4	92.1	94.8	93.5	95.3	95.0	97.9

## Data Availability

Not applicable.
